# CMV-Promoter Driven Codon-Optimized Expression Alters the Assembly Type and Morphology of a Reconstituted HERV-K(HML-2)

**DOI:** 10.3390/v6114332

**Published:** 2014-11-11

**Authors:** Oliver Hohn, Kirsten Hanke, Veronika Lausch, Anja Zimmermann, Saeed Mostafa, Norbert Bannert

**Affiliations:** HIV and Other Retroviruses, Robert Koch Institute, Nordufer 20, 13353 Berlin, Germany; E-Mails: HohnO@rki.de (O.H.); HankeK@rki.de (K.H.); LauschV@rki.de (V.L.); ZimmermannA@rki.de (A.Z.); MostafaS@rki.de (S.M.)

**Keywords:** HERV-K(HML-2), endogenous retrovirus, A-type particles, budding, HERV-K113

## Abstract

The HERV-K(HML-2) family contains the most recently integrated and best preserved endogenized proviral sequences in the human genome. All known elements have nevertheless been subjected to mutations or deletions that render expressed particles non-infectious. Moreover, these post-insertional mutations hamper the analysis of the general biological properties of this ancient virus family. The expression of consensus sequences and sequences of elements with reverted post-insertional mutations has therefore been very instrumental in overcoming this limitation. We investigated the particle morphology of a recently reconstituted HERV-K113 element termed oriHERV-K113 using thin-section electron microscopy (EM) and could demonstrate that strong overexpression by substitution of the 5'LTR for a CMV promoter and partial codon optimization altered the virus assembly type and morphology. This included a conversion from the regular C-type to an A-type morphology with a mass of cytoplasmic immature cores tethered to the cell membrane and the membranes of vesicles. Overexpression permitted the release and maturation of virions but reduced the envelope content. A weaker boost of virus expression by Staufen-1 was not sufficient to induce these morphological alterations.

## 1. Introduction

HERVs comprise over 8% of the human genome and there are more than 500,000 individual elements scattered throughout our chromosomes. These elements are derived from former exogenous retroviruses that infected germ cells or their progenitor cells and have since been vertically transferred from generation to generation [1]. HERVs are usually very ancient and are classified into different families. Indeed, members of some HERV families can be identified in Old World and New World monkeys at corresponding chromosomal loci, indicating that their integration occurred in an ancestral primate that lived at a point in time preceding the split between the groups over 35 million years ago [2,3].

The youngest (*i.e.*, the most recently integrated) retroviral elements belong to the HERV-K(HML-2) family. As they use a lysine (K) tRNA to prime their reverse transcription and are related to the mouse mammary tumor virus (MMTV), these proviruses, together with nine other families, are termed HERV-K(HML). This relationship is reflected in the acronym HML, which stands for “human MMTV-like”. Elements of the HERV-K(HML-2) family are found exclusively in Old World primates including humans. Indeed, several members are specific to humans, suggesting integration after the separation of the human and chimpanzee lineages about five million years ago.

During their residency in the host genome, endogenous retroviruses have been subjected to mutations and deletions. Purifying selection that keeps reading frames open has only been documented for a few HERV sequences [4]. The most recently integrated proviruses are therefore usually the best preserved. Currently, more than 90 well preserved HERV-K(HML-2) proviruses are known to be present in the human genome [5]. These elements still have open reading frames encoding functional viral proteins. Moreover, protein expression as well as assembly and release of non-infectious HERV-K(HML-2) particles has been observed in teratocarcinomas and melanomas and in cell lines derived therefrom [6,7]. Although not yet demonstrated, it cannot be excluded that some individuals alive today carry proviruses encoding infectious HERV-K(HML-2) elements or that infectious viruses might occasionally emerge through recombination between non-infectious elements [8]. Such recombination events reconstituting functional proviruses from inactivated endogenous elements would not be without precedence and have already been documented in animals [9,10]. A plethora of knowledge concerning the basic biology of HERV-K(HML2) and various aspects of the virus/host interaction has recently been obtained by expressing engineered consensus sequences and a reconstituted HERV-K113 element (oriHERV-K113) in which synonymous post-insertional mutations have been reverted [11,12,13].

In this current study we generated and expressed a CMV-promoter driven and partially codon-optimized version of a reconstituted HERV-K113 molecular clone (oriHERV-K113) described previously [11]. In comparison to oriHERV-K113, high expression of the codon optimized variant (termed oricoHERV-K113) lead to an unexpected switch from a C-type to an A-type-like morphology, as demonstrated by thin-section electron microscopy.

## 2. Materials and Methods

### 2.1. Plasmids

The generation of pBSK-oriHERV-K113 (“oriHERV-K113”) has been described elsewhere [11]. Briefly, the proviral sequence of HERV-K113 (GenBank AY037928) was amplified from the RP11-12X BAC-library plasmid [14] and cloned into the pBlueskript SK+ (pBSK) vector (Agilent Technologies, Inc., Cedar Creek, NE, USA). 25 non-synonymous mutations and three mutations in the 3'LTR were identified as postinsertional mutations by alignment with other HERV-K(HML-2) elements [11,15,16] and subsequently corrected by multi-site directed mutagenesis (QuikChange^®^, Agilent Technologies, Inc.). pcDNA-oricoHERV-K113 (“oricoHERV-K113”) consists of the oriHERV-K113 genome with regions codon-optimized for enhanced protein expression in mammalian cells. The complete proviral oricoHERV-K113 genome sequence was synthesized (Geneart, Regensburg, Germany) and cloned behind the CMV promoter in pcDNA3.1 (Lifetechnologies, Carlsbad, CA, USA). Construction of the human Staufen-1 expression vector has been described previously [17].

### 2.2. Cell Culture

HEK 293T and HeLa cells were cultured in complete Dulbecco’s modified Eagle medium (DMEM) supplemented with 10% fetal bovine serum, l-glutamine (2 mM), penicillin (50 U/mL) and streptomycin (50 μg/mL).

### 2.3. Virus Particle Purification and Preparation of Cell Lysates

For electron microscopy analysis and Western blots, 2.4 × 10^6^ or 5 × 10^5^ HEK 293T or HeLa cells were seeded into 100 mm dishes or 6-well plates (TPP, Trasadingen, Switzerland), respectively and subsequently transfected with the plasmids using calcium phosphate or the Qiagen Polyfect Kit (Qiagen, Hilden, Germany) according to the manufacturer’s instructions. At two to four days post transfection, samples of culture media were harvested, clarified at 3345 × g for 8 min and filtered (0.45 μm) to remove cell debris. For samples derived from 100 mm dishes, supernatants were then centrifuged at 175,000 × g for 3 h at 4 °C through a 20% sucrose cushion in a Beckman SW32Ti rotor. Virus pellets were resuspended in 60 μL of 0.05 M Hepes buffer, pH 7.2 for Western blot analysis. To prepare cell lysates, transfected cells were resuspended in cell lysis buffer (1% Triton-X 100, 20 mM Tris pH 7.7, 150 mM NaCl) containing complete protease inhibitor cocktail (Roche Diagnostic, Mannheim, Germany).

### 2.4. EM

The procedure for preparing samples for electron microscopy has been published elsewhere [15]. In short, at two days post-transfection, cells were fixed with 2.5% glutaraldehyde in 0.05 M Hepes (pH 7.2) for 1 h at room temperature before harvesting (scraping) and centrifugation at 2000 × g. Cell pellets were post-fixed with OsO_4_ (1% in ddH_2_O; Plano, Wetzlar, Germany), block-stained with uranyl acetate (2% in ddH_2_O; Merck, Darmstadt, Germany), dehydrated stepwise in graded alcohol, immersed in propylenoxide and embedded in Epon (Serva, Heidelberg, Germany) with polymerisation at 60 °C for 48 h. Ultrathin sections (60–80 nm) were cut using an ultramicrotome (Ultracut S or UCT; Leica, Germany) and stained with 2% uranyl acetate and lead citrate. For post-embedding immunostaining, cells were fixed with 4% paraformaldehyde and 0.1% glutaraldehyde. They were dehydrated by graded alcohol and embedded in Lowicryl (HM20) according to the progressive lowering temperature technique. Thin sections applied to uncoated 300hexa Mesh nickel grids were incubated on droplets of blocking buffer (5% milk powder in PBS) for 30 min, and subsequently in blocking buffer with rat anti-CA, rat anti-p15 (both polyclonal sera were prepared in the lab of the authors) or goat anti-GFP (Rockland Immunochemicals for Research, Limerick, PA, USA) and a 5 nm gold-conjugated anti-rat/goat antibody (BB International, Cardiff, UK) for 1 h. After fixation with 0.1% glutaraldehyde in 0.05% Hepes buffer for 15min, samples were counterstained with 2% uranyl acetate and 0.2% lead citrate. Transmission electron microscopy was performed with an EM 902 (Zeiss) operating at 80 kV and the images were digitized using a slow-scan charge-coupled-device camera (Pro Scan, Scheuring, Germany).

### 2.5. SDS PAGE and Western Blot Analysis

Viral lysates or pelleted virus particles were mixed with 5× sample buffer and heated for 10 min at 98 °C before being subjected to sodium dodecyl sulphate-polyacrylamide gel electrophoresis (SDS-PAGE). The proteins were transferred onto a PVDF membrane (Roth, Karlsruhe, Germany) by semi-dry electrophoresis (Biorad, München, Germany). After blocking in PBS with 5% skim milk powder and 0.1% Tween, the membranes were probed with specific rat anti-CA sera as described previously [15] and a secondary horseradish peroxidase-conjugated goat-anti-rat antibody (Sigma-Aldrich, Hamburg, Germany). The β-actin was detected with the HRP-conjugated AC-40 mouse monoclonal antibody (Sigma-Aldrich). Proteins were visualized using SuperSignal West Dura Chemiluminescent Substrate (Thermo Fisher Scientific Inc., Waltham, MA, USA) and Kodak Medical X-ray film.

## 3. Results

### 3.1. Generation of a Codon-Optimized Molecular Clone of a Reconstituted HERV-K(HML-2) Element

We previously described 25 non-synonymous post-insertional mutations in the HERV-K113 provirus originally characterised by Turner and co-workers [11,14,15,16]. Reversion of these 25 mutations and the reversion of three presumed additional post-insertional mutations in the 3'LTR of a molecular clone (to yield a clone termed oriHERV-K113) allowed the expression of replication-defective retroviral particles in transfected human cell lines [11].

**Figure 1:**
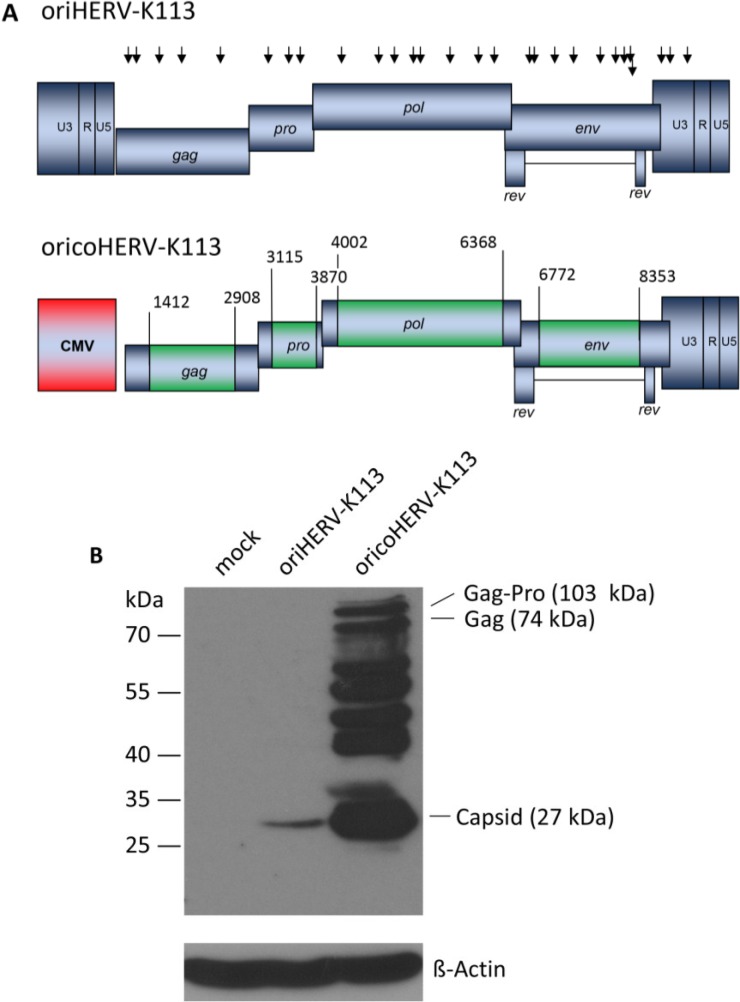
Genomic organization and expression of the reconstituted human endogenous retrovirus (HERV-K) human MMTV-like-2 HERV-K(HML-2) sequence “oriHERV-K113” and the partial codon-optimized variant with CMV promoter “oricoHERV-K113”. (**A**) For reconstitution of the potential infectious progenitor sequence of the HERV-K(HML-2) element, 25 non-synonymous putative post-insertional mutations (indicated by black arrows, lower arrow refers to *rec* reading frame) in the HERV-K113 genome sequence (AY037928) were reverted to generate oriHERV-K113. In oricoHERV-K113, parts of the oriHERV-K113 genome were codon-optimized (boxed in green) to achieve maximal expression in mammalian cells, leaving potential regulatory sequences or overlapping regions in the genome unaltered. The 5'LTR region was replaced by a CMV promoter (red). Numbers given for start and end of codon-optimized regions refer to genbank sequence AY037928. (**B**) Western blot of intracellular Gag proteins, processing fragments and degradation intermediates after transfection of the molecular clones in HEK 239T cells using a Gag (CA)-protein-specific antiserum for detection. Bands presumably representing the Gag-Pro, Gag and CA proteins are indicated. Stripping and re-probing of the membrane with ß-Actin specific monoclonal antibody demonstrated that the same amounts of cell lysate were loaded for mock, oriHERV-K113 and oricoHERV-K113 transfected cells, respectively.

In order to boost the relatively low levels of particle expression, we also constructed a molecular clone in which parts of the oriHERV-K113 genome were codon-optimized, leaving potential regulatory sequences and regions of overlapping reading frames unchanged [15]. To further enhance expression, we replaced the viral 5'LTR with a CMV promoter and named the resulting near-full-length molecular clone “oricoHERV-K113” (Figure 1A).

Western blot analysis of transfected HEK 293T cells using a capsid-specific antiserum revealed that Gag expression from oricoHERV-K113 was at least 50-fold higher than that of oriHERV-K113 (Figure 1B).

### 3.2. Differences in the Morphologies of oriHERV-K113 and oricoHERV-K113

We next set out to analyze the morphological impact of the modulated expression of retroviral proteins by thin-section EM analysis of human cell lines transfected with oricoHERV-K113.

**Figure 2:**
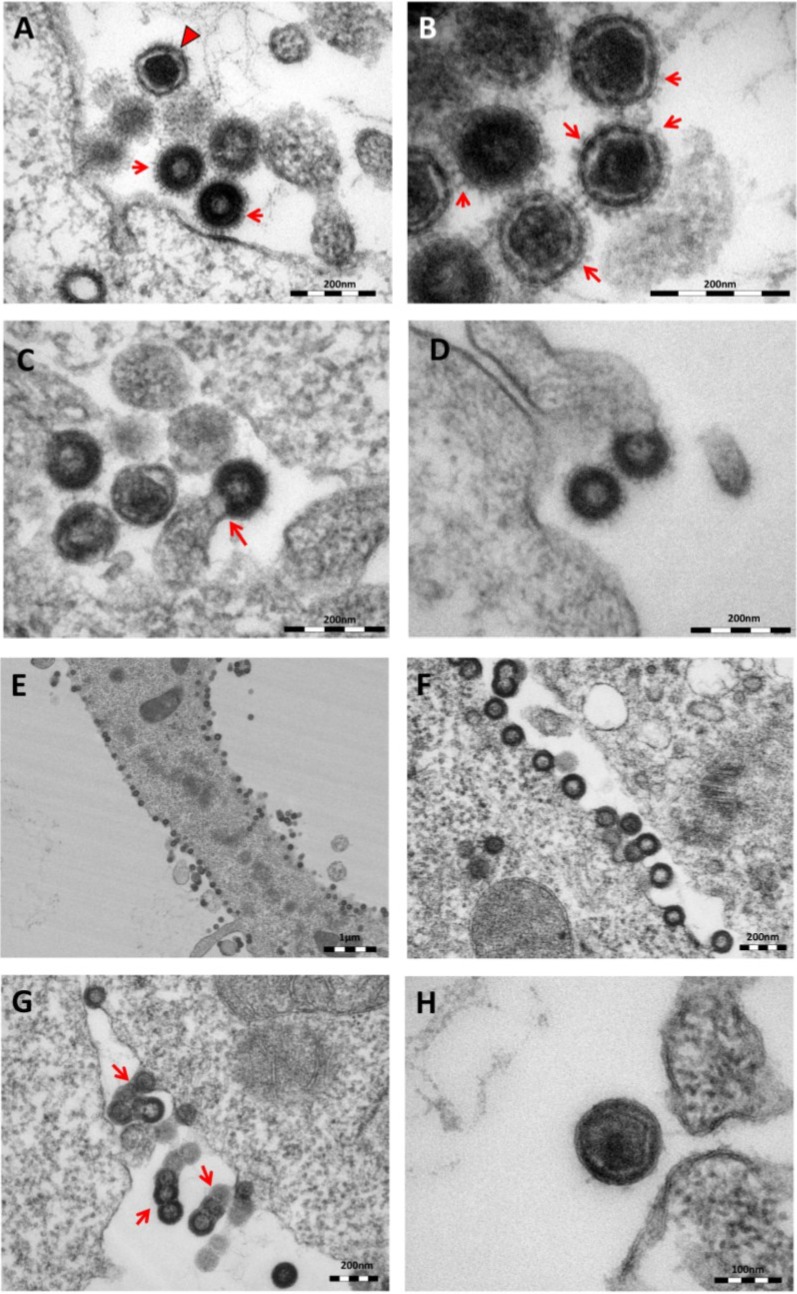

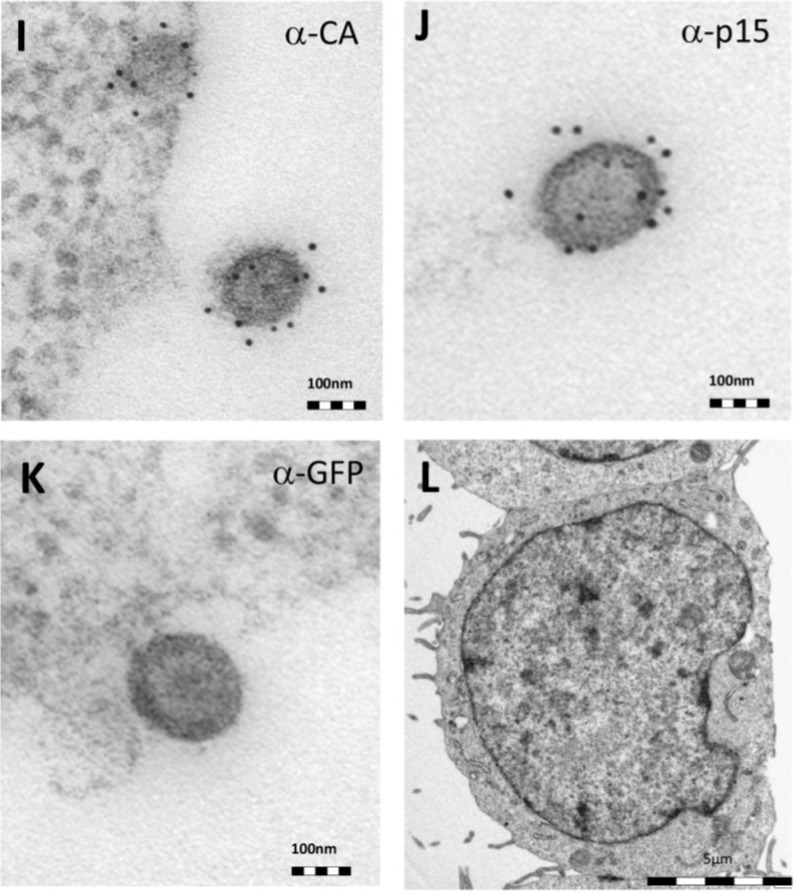
Thin-section electron micrographs of oriHERV-K113 and oricoHERV-K113 particle production after transfection with full-length molecular clones. (**A**) In HEK 293T cells, both immature oriHERV-K113 derived particles with ring-like structures of Gag proteins (marked by arrows) and mature forms with the condensed cores (marked by an arrowhead) are seen. (**B**) Env is incorporated strongly into the viral membrane (arrows). (**C**) Budding of oriHERV-K113 particles is of typical C-type morphology with the assembly of the Gag shells at the cellular membrane during budding (an arrow indicates a budding stage). (**D**) oriHERV-K113 particles produced in HeLa cells. (**E**,**F**) oricoHERV-K113 transfected HEK 293T cells produce large numbers of particles that line up with the cellular membrane and display an A-type morphology, predominantly with preassembled rings of Gag protein attached to the membranes. (**G**) oricoHERV-K113 occasionally forms budding chains with structures resembling C-type morphology (arrows). (**H**) Incorporation of Env into the viral membrane of oricoHERV-K113 appears to be very low. Shown is a released mature particle produced by HeLa cells (**I**) Immunostaining EM with a CA specific antiserum. (**J**) Immunostaining EM with a p15-specific antiserum and a GFP control antiserum (**K**). (**L**) No transfection control.

As previously reported [11], oriHERV-K113 produces particles with immature and condensed mature cores and many envelope spikes clearly visible at the viral membrane (Figure 2A,B).

The overall production of viral particles is low, with only few budding stages or clusters of released immature and mature particles per cell. Cell sections with oriHERV-K113 virions were relatively rare. Only about one out of 20–30 cell sections contained particles. The budding morphology of oriHERV-K113 is typically C-type, despite it being a betaretrovirus according to its *pol* region sequence [18]. The Gag molecules commence assembly at the cell membrane in a convex formation that resembles in form the letter “C” (Figure 2C). We never observed a complete closure of the Gag ring during the late budding stages, indicating a virtually concomitant completion of the Gag shell and pinching off of the virion. There were also no immature virus-like particles present in the cytoplasm of cells transfected with oriHERV-K113. The results following transfection of the oriHERV-K113 full-length molecular clone into HeLa cells were similar to those with HEK 293T cells (Figure 2D).

The picture is different in cells transfected with oricoHERV-K113. In these cells, particles with completely closed Gag shells line up at the inner leaflet of the plasma membrane (Figure 2E,F) and in some cells also at the cytoplasmic side of vesicular membranes. The cytoplasmic assembly of particles having completely closed immature cores is reminiscent of the regular retroviral A- and B-type morphology and is comparable to the mode of assembly of typical betaretroviruses including mouse MMTV and Mason Pfizer Monkey Virus (MPMV) [19]. Many of the transfected cells display budding chains in plasma membrane protrusions (Figure 2G). Although intracellular particles predominate, immature and mature virions are also present outside of the cells (Figure 2G,H), indicating some degree of particle release from the cell. The incorporation of Env in the viral membrane appears to be significantly lower compared to that in particles produced by oriHERV-K113, with very few spikes being present on budding structures or released particles (Figure 2G,H). Expressing oricoHERV-K113 in HeLa cells results in morphologies similar to those observed in HEK 293T cells (Figure 2H). To confirm the identity of the oricoHERV-K(HML-2) particles we have performed immunostaining EM on thin-sections of transfected HEK 293T cells embedded in Lowicryl. Intra- and extracellular viral particles were specifically labelled with a CA and p15 specific antiserum, but not with an anti-GFP control (Figure 2I–K) No virus particles were observed in untransfected cells (Figure 2L).

**Figure 3:**
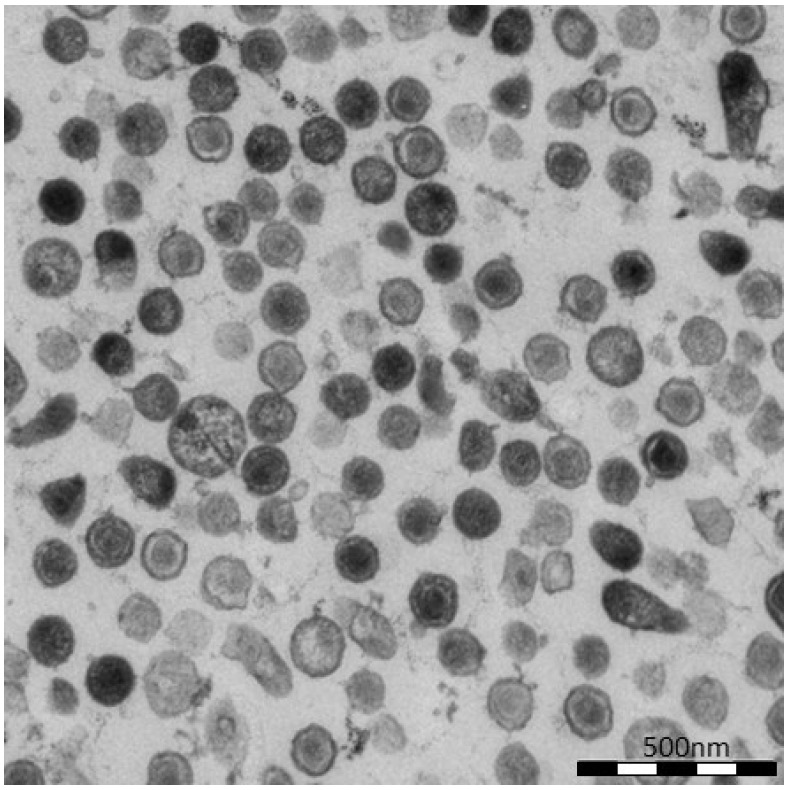
Virus particles in the supernatant of transfected HEK 293T cells. Cells were transfected with the full-length molecular clone oricoHERV-K113 and virions in the supernatant were pelleted by ultracentrifugation and embedded for thin-section EM. Nearly all particles show the condensed core of mature virions.

To further demonstrate virion release from cells transfected with oricoHERV-K113, the supernatant of transfected HEK 293T cells was pelleted by ultracentrifugation and embedded for thin-section EM. As shown in Figure 3, the pellets contain high proportions of exclusively mature virions with relatively large, typically concentric spherical or polygonal electron dense core structures against a background of contaminating microsomes (Figure 3). In agreement with the observations made in the previous experiment, spikes are barely visible on the surface of oricoHERV-K113 particles. Our data therefore demonstrate the efficient release and maturation of retroviral particles from cells transfected with oricoHERV-K113.

### 3.3. Enhancement of oriHERV-K113 Expression by Staufen-1 Overexpression Does Not Result in a Morphology Switch from C- to A-Type

We recently described the interaction of the HERV-K(HML-2) encoded proteins Rec and Gag with the human Staufen-1 protein and noticed a 20-fold increase in oriHERV-K113 production in cells overexpressing Staufen-1 [17].

**Figure 4:**
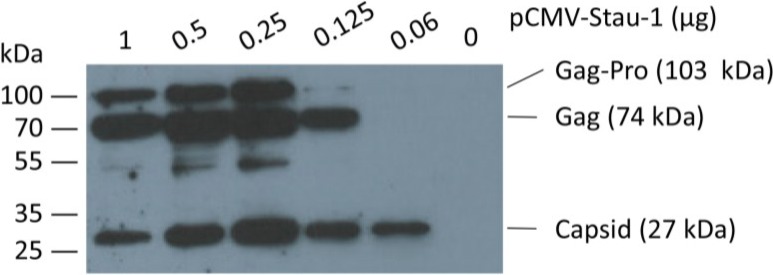
Co-expression of the human Staufen-1 protein enhances oriHERV-K113 protein production. Western blot detection by using a Gag (CA)-specific antiserum of intracellular HERV Gag protein 48 h after co-transfection of HEK 293T cells with a fixed amount of oriHERV-K113 full-length molecular clone and decreasing amounts of the Staufen-1 expression vector. The presumed Gag-Pro, Gag and processed capsid proteins are indicated. The exposure time was optimized to show expression differences. It was too short to visualize Gag proteins on lane 6 (no Staufen-1 overexpression).

In order to boost oriHERV-K113 expression by Staufen-1 as strongly as possible and to evaluate a potential switch in morphology, we first optimized the transfection ratio of oriHERV-K113 and Staufen-1 expression plasmids. As shown in Figure 4, the peak of Gag expression is associated with an intermediate amount of Staufen-1 DNA; *i.e.*, with a 4:1 ratio of oriHERV-K113 to Staufen-1 DNA.

**Figure 5:**
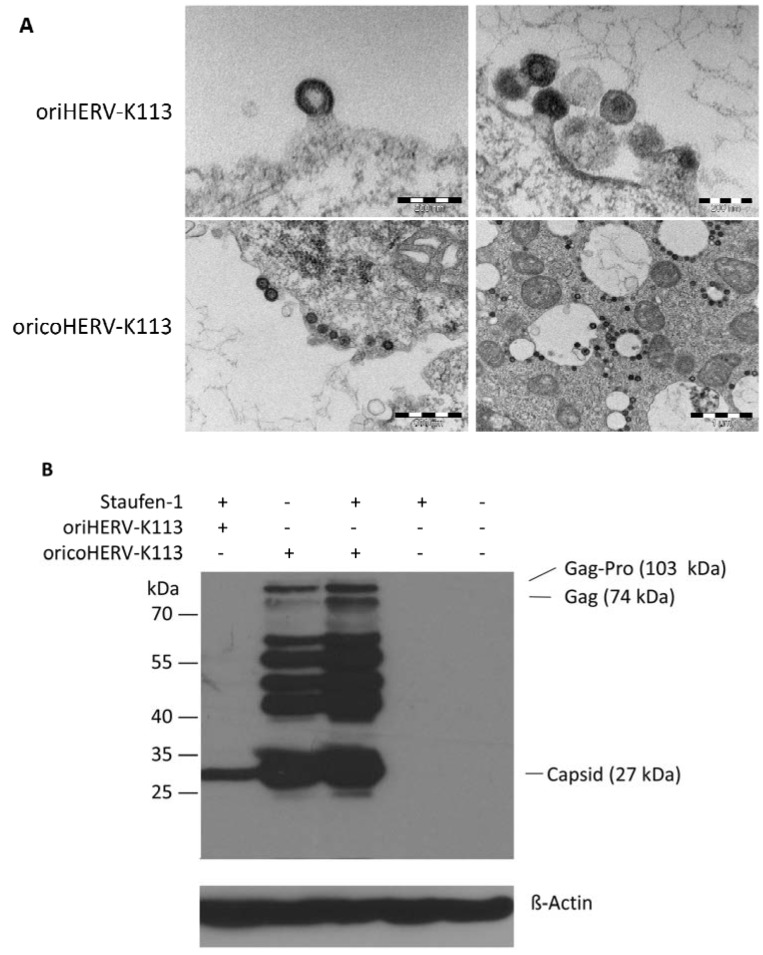
Thin-section EM of oriHERV-K113 and oricoHERV-K113 particle production after co-transfection of full-length molecular clones with Staufen-1 in a 4:1 ratio to enhance protein production. (**A**) Upper panel: EM of particles produced by oriHERV-K113 after co-transfection with Staufen-1. No changes in C-type morphology can be seen despite enhancement of protein production by Staufen-1. Lower panel: Same experiment with oricoHERV-K113, no influence on the A-type morphology can be seen as a result of moderate Staufen-1 enhancement. (**B**) Western blot of Staufen-1-mediated enhanced Gag protein expression in HEK 293T cell lysate. Cells were transfected with oriHERV-K113 and Staufen-1 (4:1 ratio), oricoHERV-K113, oricoHERV-K113 and Staufen-1 (4:1 ratio), Staufen-1 alone and with empty vector as mock control (left to right). Intracellular Gag was detected by Gag (CA)-protein specific antiserum. As loading control, the membrane was stripped and re-probed with a ß-Actin specific monoclonal antibody.

The morphology of viral particles produced by transfected HEK 293T cells under optimized Staufen-1 mediated enhancement conditions were also analyzed by thin-section EM. Despite Staufen-1 mediated enhancement resulting in significantly more viral particles, there was no evidence for a C- to A-type switch (Figure 5A, upper panel). However, even when enhanced by Staufen-1, the levels of Gag expression were still lower compared to that in cells transfected with oricoHERV-K113 (Figure 5B). As expected, co-transfection of Staufen-1 did not alter the morphology of oricoHERV-K113 particles (Figure 5A, lower panel) and had only a minimal enhancing effect on the expression of the oricoHERV-K113 Gag protein (Figure 5B), which is probably due to the high baseline level of protein expression from this construct.

## 4. Discussion

Codon-optimization of retroviral sequences is commonly used to enhance protein expression in eukaryotic cells [20,21]. For the structural viral proteins, this is achieved in two main ways: First, it increases the efficiency of translation at the ribosome and, second, it releases transcripts from the nuclear restrictions involved in the control of nucleocytoplasmic transport of unspliced or partially spliced viral mRNAs. In an attempt to boost the expression of oriHERV-K113, a reconstituted HERV-K(HML-2) element, we codon-optimized large sections of the genes coding for Gag, Pro, Pol and Env. Regions containing the presumed packaging site, slippery sites, splice sites and the Rec-responsive element were left unchanged [15]. Additionally, the relatively weak 5'LTR promoter was substituted for the stronger CMV promoter. The resulting construct was termed “oricoHERV-K113”.

Upon transfection into eukaryotic cells, a dramatic increase in the levels of cytoplasmic Gag was detected compared to those in cells transfected with oriHERV-K113. Interestingly, in oricoHERV-K113 transfectants, thin-section electron microscopy revealed the presence of a multitude of cytoplasmic particles with apparently completely assembled Gag shells. Such intracellular particles with immature cores had never been seen in cells expressing oriHERV-K113, where only budding stages or released immature and mature virions were seen. This is consistent with earlier observations in teratocarcinoma cell lines [6,22] and with the documented morphologies of virions or virus like particles generated with HERV-K(HML-2) consensus sequences [12,13]. In these reports, particles budded at the cell membrane with a conventional C-type morphology [23,24] even if the transcription of the consensus sequences was driven by a CMV-promoter [12,13]. Based on these observations we speculate that the CMV-promoter alone would not be sufficient to cause formation of closed immature intracellular HERV-K113 cores. Whether this is true or whether codon optimization is already sufficient to induce intracellular particles needs to be resolved in experiments with appropriate vectors. Initially, retroviruses were classified according to their appearance and budding phenotype as visualized by EM [23,24] (reviewed by [25]). The C-type morphology is typically associated with oncoviruses and lentiviruses where formation of the immature core occurs concomitantly with budding at the cell membrane. In the case of B- and D-type retroviruses, the assembly of immature capsids occurs in the cytoplasm with subsequent transport to the cell membrane. Furthermore, completely assembled immature cytoplasmic cores of the prototype B-type retrovirus MMTV are designated as A-type particles [24]. Unusual groups in this respect are the intracisternal A-type particles of rodent cells that are encoded by endogenous elements and which assemble and bud into cisternae of the endoplasmic reticulum [26]. With respect to morphology, the highly abundant oricoHERV-K113 particles are best classified as A-type. The vast majority of the particles are tethered to the plasma membrane or to the membranes of vesicular structures. Budding chains are very frequently seen with incomplete immature cores, although regular budding stages are also occasionally observed. Taking these observations into account, we assume that the A-type particles of oricoHERV-K113 are assembled at membranes. Whether the assembly sites are confined to the plasma membrane or whether assembly also takes place at vesicular membranes remains unknown. The type A-particles clearly present on the outside of vesicles might be explained by passive transport during endocytotic processes. The low frequency of viral particles inside of these vesicles that are besieged by virions indicates that budding into the lumen does not occur or is very inefficient.

In contrast to the situation with vesicles, the release of oricoHERV-K113 virions into the supernatant is very efficient. Thin-section EM of virus pellets from supernatants of oricoHERV-K113 expressing cells reveals high numbers of particles. It is, however, currently unclear to which extent type A-particles are able to leave the cell or whether the pelleted particles were released by C-type budding occurring in parallel to the formation of A-type particles. Whatever the dominant budding phenotype, the particles released have mature cores. Efficient egress and maturation of oricoHERV-K113 has been confirmed by Western blotting and the measurement of RT activity in the supernatants (data not shown). These findings indicate that the partial codon-optimization of oriHERV-K113 does not prevent the switching of reading frames, the production of Gag-Pro and Gag-Pro-Pol precursor proteins or the release of active protease and reverse transcriptase. These results are consistent with those we obtained earlier by expressing the Gag-Pro-Pol region of oricoHERV-K113 alone [15]. The use of a strong promoter and partial codon optimization of oricoHERV-K113 did not enhance expression of Env to the same degree seen for the Gag-polyproteins. As a consequence, spikes on oricoHERV-K113 particles are rarely observed. Whether the level of Env expression or the cellular Env to Gag ratio plays a role in the formation of HERV-K(HML-2) type A particles needs to be determined and is currently under investigation.

Despite a significant boost, it was not possible to drive oriHERV-K113 into the production of type A-particles by overexpression of Staufen-1, a binding partner of the Rec protein [17]. However, the enhanced levels of Gag expression were still significantly lower than those seen with oricoHERV-K113 alone. We therefore favor the hypothesis that a critical level of expression required for the production of HERV-K(HML-2)-derived type A-particles was not reached.

Whether this type of particle is formed *in vivo* and whether they have a relevant biological role is also a matter of speculation. It is conceivable in this context that such cores might be potentially transferred from cell-to-cell or might play a role in retrotransposition events contributing to HERV-K(HML-2) proliferation following endogenization. Whatever the biological role and the underlying molecular mechanism of the morphological switch reported in this paper is, it is certain that EM will continue to play a pivotal role in future studies aimed at shedding light on this issues.
